# 

**Published:** 1871-02

**Authors:** 


### CONTENTS.

COMMUNICATIONS.

Nervous System .... 53
Dental Hygiene .... 57
Office Notes .... 61

PROCEEDINGS OF SOCIETIES.

Fifth Annual Meeting of the Ohio Dental Society .... 64
Proceedings of the Second District Dental Society, N. Y .... 82

SELECTIONS.

Boston Society of Medical Sciences .... 86
Preservation of Anatomical Specimens .... 88
Rhode Island Medical Society .... 90
Hygienic Treatment of Disease .... 90
Deaths from Chloroform .... 91
Speaking and Singing without a Tongue .... 91
Eau de Beaute de M. Bargasse .... 92
New Test for Arsenic .... 92

EDITORIAL.

Professional Responsibility .... 93
How not to do it .... 94
Pneumatic Engine .... 95

